# A Light-Driven Self-Spinning and Translation Disc Exploiting Photothermal Liquid Crystal Elastomers

**DOI:** 10.3390/mi17030284

**Published:** 2026-02-25

**Authors:** Cong Li, Leyi Xu, Yuntong Dai, Yu Dai

**Affiliations:** School of Civil Engineering, Anhui Jianzhu University, Hefei 230009, China; xuleyi0826@163.com (L.X.); daiytmechanics@ahjzu.edu.cn (Y.D.); 15839775882@163.com (Y.D.)

**Keywords:** self-spinning, liquid crystal elastomer, disc, light-driven

## Abstract

Self-sustained oscillatory systems enable autonomous motion through continuous interaction with ambient energy sources, positioning them as promising candidates for soft robotic actuation, energy conversion, and biomedical applications. However, their utility is often limited by inherent vibrations and frictional losses, which can lead to impaired efficiency and generate noise. To overcome these limitations, a continuously rotating disc mechanism is proposed, which exploits the photothermal response of liquid crystal elastomers (LCEs) under uniform illumination. The resulting temperature field within the material is obtained via photothermal modeling of the LCE. The rotational actuation torque is generated through mass displacement resulting from light-induced LCE contraction. Based on the above conditions, we establish the equilibrium conditions and critical thresholds for continuous motion and reveal a synergy between the thermal field and torque. Through the interplay of the temperature field and the actuating rotating moment, the system ultimately attains steady self-rotation. Therefore, the absorbed energy offsets damping losses. Numerical simulations reveal that the steady-state self-spinning and translational velocity are influenced by multiple parameters including incident heat flux, gravitational field strength, material contraction coefficient, LCE element dimensions, illumination geometry, and resistive torque. The proposed LCE disc configuration exhibits exceptional operational stability and minimal damping, which has potential for implementation in advanced soft robotic systems and mechanical energy conversion applications.

## 1. Introduction

Self-oscillating systems maintain cyclic motion by drawing energy from a normal environment [1,2,3]. This feature is fundamentally passive. Rather than relying on complex internal drivers, such systems harness their surroundings to sustain oscillations—an approach that simplifies design and elevates overall efficiency [4,5,6]. Notably, self-sustained oscillatory behavior can be maintained without periodic external excitation; instead, continuous motion is sustained solely by a constant energy supply, significantly reducing the need for complex control strategies. By continuously absorbing environmental energy, these systems maintain cyclic behavior autonomously. The period and amplitude of the oscillatory motion are primarily controlled by the system’s inherent characteristics, endowing it with strong robustness [7,8].

The liquid crystal elastomers (LCEs) are excellent materials to drive self-oscillating systems. LCEs are a unique class of intelligent materials whose molecular structure consists of a cross-linked polymer network integrated with oriented mesogenic units [9,10]. This unique composition endows LCEs with dual characteristics, the high elasticity and large deformation capability of rubbery polymers [11,12,13]. Owing to the anisotropic properties of liquid crystals under external stimuli such as temperature [14,15], light [16,17], or electric [18,19] fields, the orientation of the mesogenic can change, which leads to reversible shape transformations in the material [20,21,22,23]. These features render self-sustained oscillatory systems particularly attractive for applications involving energy conversion [24,25,26], soft robotics [27,28,29,30], medical technologies [31,32], and micro-nano devices [33,34]. In this study, a pre-aligned monodomain LCE is assumed to ensure deterministic anisotropic photothermal contraction, which is essential for generating directional torque and is not achievable with isotropic conventional elastomers.

Despite outstanding progress, many self-oscillating LCE systems still face a common challenge: inherent oscillations, friction, and associated noise [35,36]. These issues stem from the driving system being cyclical and periodic—such as bending [37,38], rolling [39,40], vibration [41,42], jumping [43,44], and chaos [45,46,47,48]—which can result in reduced energy efficiency, material fatigue, and operational instability [49,50]. Because damping-induced energy dissipation cannot be avoided during operation, structural feedback mechanisms leveraging active materials have recently been proposed to compensate for these losses, such as the coupling between chemical reactions and large mechanical deformations [51,52,53], self-shadowing effects [54], and coupled multi-stage processes driven by liquid evaporation [55]. These mechanisms are characterized by nonlinear interactions and feedback dynamics [56,57], forming the essential basis of self-sustained oscillation. LCEs can be driven by various methods, including photochemical, thermal [58,59,60], electrical, and magnetic drives [61,62]. Compared with other driving methods, LCEs exhibit pronounced performance benefits under photothermal stimulation, especially in the visible-light regime, which makes them well suited for photomechanical actuation [63,64,65,66]. In contrast to electrically driven systems, light-driven approaches enable wireless operation without external power sources [67], thereby decreasing the overall device mass. Light-driven systems enable non-contact actuation, thereby increasing operational flexibility and broadening their range of potential applications [68,69,70,71]. However, many self-oscillating LCE systems still face some common limitations, such as low energy efficiency and compromised stability.

In recent years, numerous theoretical and experimental studies have investigated photothermal actuation and temperature distribution in liquid crystal elastomer systems. Several previous works have proposed analytical models describing thermal fields and deformation behaviors under asymmetric illumination, which provide valuable foundations for understanding LCE-driven motion [72]. However, most of these studies primarily focus on oscillatory, bending, or rolling behaviors induced by periodic excitation or feedback mechanisms, and comparatively fewer efforts have addressed steady continuous rotational motion under constant illumination.

To address the limitations of conventional systems, a new paradigm shifting from oscillatory to steady hybrid motion is required. This work presents a light-driven system based on an LCE disc capable of sustained spinning and continuous translation on dual tracks. Through systematic theoretical modeling, the mechanisms behind the photothermally driven torque and propulsive force, and how key parameters—such as heat flux, material contraction coefficient, structural dimensions, and illumination zone—govern the motion characteristics, are systematically revealed. The present study provides a novel design strategy and theoretical foundation for developing new soft actuators that are quiet, efficient, and wirelessly controlled, demonstrating broad application prospects in fields requiring high silence and stability, such as precision manipulation, covert reconnaissance, and biomedical devices.

The manuscript is organized as follows. Section 2 elaborates on the theoretical model, including temperature field generated under asymmetric illumination. Section 3 investigates the light-induced rotational torque resulting from centroid shift and analyzes the influence of key system parameters on self-spinning and translational velocities. In Section 4, the dependence of the steady angular velocity on system parameters is analyzed. Finally, Section 5 summarizes the principal findings of the study and explores the potential real-world applications of the proposed system.

## 2. Model and Theoretical Formulation

In this section, a light-driven self-spinning and translation of a liquid crystal elastomer disc system is introduced, which consists of an LCE disc, a shading film and dual-track. And the steady-state temperature field of the LCE under coupled spinning and translational motion is characterized through photothermal modeling. Eventually, the formula for the driving torque can be obtained.

### 2.1. Temperature Distribution of a Steadily Rotating LCE-Based Disc

**Figure 1:**
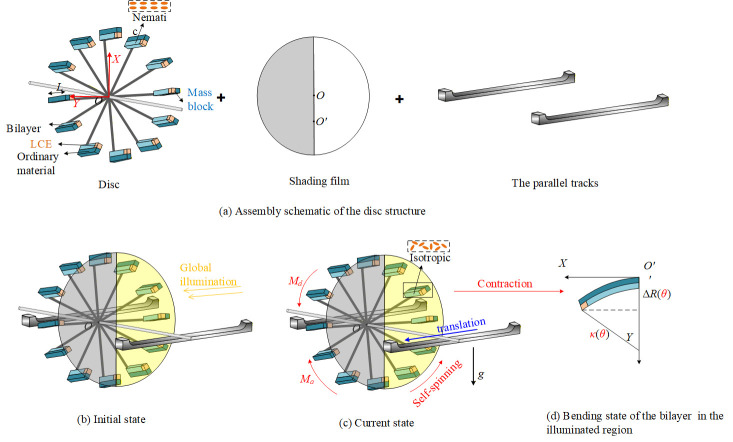
The structure of the LCE-based disc.

The schematic diagram of the assembled LCE-based rounded disc structure, which is designed for self-spinning and translation under steady global illumination, is shown in Figure 1. The system is composed of multiple bilayer elements mounted on a central disc, along with parallel tracks arranged on both sides. A light-shading film, matching the disc in shape, is affixed to its front. The left half of the film is opaque, while the right half is transparent. The film’s center of mass is deliberately positioned lower, thus ensuring its orientation remains unaffected by the disc’s motion. Each bilayer unit consists of an LCE layer and a passive substrate, equipped with a mass block at the end that significantly increases the system’s rotational inertia. The geometric parameters, including the bilayer length (*L*) and disc radius (*R*), are defined in Figure 1a. Assuming that the mass of each bilayer is negligible compared with that of the attached block, its inertial contribution is neglected. A key aspect of the design is the restricted illumination zone, which is confined to the right half of the disc. This region, subjected to uniform and constant collimated light normal to the plane to avoid self-shadowing among bilayers, is highlighted in yellow in Figure 1b.

Within the illuminated region on the right side, as seen in Figure 1b, the LCE layers undergo bending, whereas the LCE layers in the non-illuminated region on the left side remain in their original configuration. This spatial asymmetry between the two sides of the disc breaks the rotational symmetry of the mass distribution, leading to a continuous displacement of the center of mass relative to the geometric center, which in turn drives self-spinning and translational motion. Entry into the illuminated region induces light absorption in the LCE layer, triggering a nematic–isotropic phase transition. The resulting photothermal response causes unequal longitudinal contraction between the active LCE layer and the passive layer, leading to downward bending of the bilayer. Consequently, the additional mass block is displaced from its initial radial location, as shown in Figure 1d, inducing the system’s center of mass to deviate from the rotational axis. Subjected to the gravitational force, the resulting off-center mass produces an actuating torque that initiates and maintains the rotation of the disc. After exiting the illuminated region, the LCE layer shifts back to the nematic phase where it cools gradually, enabling the bilayer to return to its planar configuration and the mass to revert to a position close to its original location. With increasing rotational speed, a dynamic equilibrium is attained as illumination and recovery cycle repeatedly. Finally, the system stabilizes into a steady self-spinning and translation regime at a given angular velocity, in which the time-averaged driving torque is counterbalanced by dissipative damping.

Under constant and asymmetric illumination, the LCE’s bilayer arms undergo localized photothermal actuation. Specifically, bilayer arms within the illuminated region exhibit light-induced bending, while those in the non-illuminated region maintain their original configuration. This spatially non-uniform actuation breaks the rotational symmetry of the system’s mass distribution, resulting in a continuous translational shift in the center of mass relative to the geometric center—a motion synchronized with the system’s self-spinning.

The gravitational load acting on this off-center mass can be decomposed in the system’s reference frame. Crucially, the projection of the resulting gravitational component along the tangential (orbital) direction exhibits an asymmetric spatial distribution. In the front half of the self-spinning, the tangential projection of gravity on the bent bilayer arms generates positive thrust. In the rear half, it produces negative resistance. The persistent imbalance between thrust and resistance accumulates into a steady net driving force. This force acts on the entire disc system, overcoming frictional losses and other damping effects, ultimately initiating and sustaining stable forward orbital motion.

As illustrated in Figure 1c, the LCE-based disc is subjected to constant and uniform light. Since the thickness of the LCE layer is negligible compared with the optical penetration depth, a uniform temperature distribution is assumed at any given angular location. Under steady self-spinning conditions, the LCE layers undergo thermal exchange with the surrounding environment. Under the operating conditions, the small characteristic thickness ensures that the Biot number is less than one. Under these conditions, temperature variation across the thickness can be neglected, and the inherent oscillations, friction, and associated noise are not considered, thereby validating the lumped-temperature assumption [64,73,74,75,76] employed in our model. Consequently, the following equation can be used to characterize the temperature field,

(1) $$\frac{dT\left(\theta ,t\right)}{dt}+\vec{V}\left(t\right)\nabla T\left(\theta ,t\right)=\frac{I\left(\theta \right)-KT\left(\theta ,t\right)}{\rho_{c}}$$

where $\rho_{c}$ is the specific heat capacity, θ is the angular coordinate along the disc circumference, K is the heat transfer coefficient, $I\left(\theta \right)$ is the heat flux at any angular position of the LCE layers, $\vec{V}\left(\theta ,t\right)=\omega R\vec{e_{\theta }}$ is the velocity field of the LCE-based disc at time *t*,ω is the angular velocity, $\vec{e_{\theta }}$ is the unit vector along the tangential direction, and $\nabla T\left(\theta ,t\right)$ is the gradient of $T\left(\theta ,t\right)$.

When the system is in steady self-spinning and translation, ω remains fixed, producing a velocity field characterized by uniform magnitude and direction. Assuming that the temperature distribution depends only on the θ and t.

To obtain an analytic solution for Equation (1), we define the following activation function,

(2) $$I\left(\theta \right)=\frac{I_{0}}{2}-\frac{I_{0}}{2}\mathrm{sigmod}\left[k\left(\theta -\theta_{1}\right)\left(\theta -\theta_{2}\right)\right]$$

where $I_{0}$ corresponds to the heat flux resulting from uniform light exposure, while k is the transition parameter that governs how $I_{0}$ changes across the boundary between illuminated and dark zones. The illuminated region is on the right side, as seen in Figure 1b, so $\theta_{1}=\frac{\pi }{2}\;\mathrm{and}\;\theta_{2}=\frac{3\pi }{2}$.

Once the disc attains a steady state of simultaneous self-spinning and translation, $T\left(\theta ,t\right)=T\left(\theta \right)$, Equation (1) can be rewritten as,

(3) $$\omega \frac{dT\left(\theta \right)}{d\theta }=\frac{I_{0}-I_{0}sigmod\;\left[k\left(\theta -\theta_{1}\right)\left(\theta -\theta_{2}\right)\right]-2KT\left(\theta \right)}{2\rho_{C}}$$

**Figure 2:**
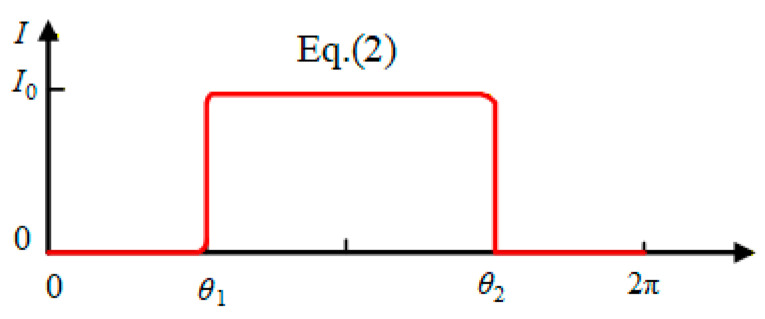
Heat flux continuity $I\left(\theta \right)$ as defined in Equation (2).

Let k=200, $\alpha =\left[\theta_{1},\theta_{2}\right]$, $I\left(\theta \right)=I_{0}$ inside the illuminated zone, and $\alpha =\left[0,\theta_{1}\right]⋃\left[\theta_{2},2\pi \right]$,$\;I\left(\theta \right)=0$ outside the illuminated zone, as shown in Figure 2. The value of k determines how $I\left(\theta \right)$ transitions within the transitional region of the light and dark zones. Let $\bar{\omega }=\omega T_{0}$, $\bar{I_{0}}=I_{0}/KT_{e}$, $\bar{T}=T/T_{e}$ and $\tau_{0}=\rho_{c}/K$ ($\tau_{0}$ represents the thermal time constant), the following forms can be derived from Equations (2) and (3),

(4) $$\bar{I}\left(\theta \right)=\frac{\bar{I_{0}}}{2}-\bar{\frac{I_{0}}{2}}sigmod\left\{k\left[\theta -\theta_{1}\right]\left[\theta -\theta_{2}\right]\right\}$$

and

(5) $$\bar{\omega }\frac{d\bar{T}\left(\theta \right)}{d\theta }=\frac{\bar{I_{0}}}{2}-\frac{\bar{I_{0}}}{2}sigmod\left\{k\left[\theta -\theta_{1}\right]\left[\theta -\theta_{2}\right]\right\}-\bar{T}\left(\theta \right)$$

As a result,

(6) $$\bar{T}\left(\theta \right)=e^{-\frac{\theta }{\bar{\omega }}}\left[c_{1}+\frac{\bar{I_{0}}}{2\bar{\omega }}\int_{0}^{\theta }e^{\frac{\delta }{\bar{\omega }}}\left\{1-sigmod\left[k\left(\delta -\theta_{1}\right)\left(\delta -\theta_{2}\right)\right]\right\}d\delta \right]$$

The boundary condition is $\bar{T}\left(0\right)=\bar{T}\left(2\pi \right)$, and the undetermined coefficient $c_{1}$ is derived as shown in the following expression:

(7) $$c_{1}=\frac{{\bar{T}}_{0}}{2\bar{\omega }}\cdot \frac{e^{-\frac{2\pi }{\bar{\omega }}}}{1-e^{-\frac{2\pi }{\bar{\omega }}}}\int_{0}^{2\pi }e^{\frac{\delta }{\bar{\omega }}}\left\{1-sigmod\left[k\left(\delta -\theta_{1}\right)\left(\delta -\theta_{2}\right)\right]\right\}d\delta$$

Furthermore, $\bar{T}\left(\theta \right)$ can be derived.

### 2.2. Light-Induced Driving Self-Spinning and Translational Torque by Gravity

It is assumed that the mass of the attached blocks is substantially larger than that of the bilayers during self-spinning and translation. Thus,

(8) $$M_{a}=M_{a1}+M_{a2}$$

Here, $M_{a1}$ is defined as the driving torque due to the disc’s self-spinning, and $M_{a2}$ is the driving torque arising from its orbital translation.

Clearly,

(9) $$M_{a1}=\frac{mg}{4\pi }\int_{\pi /2}^{3\pi /2}\left[R-∆R\left(\theta \right)\right]sin\theta d\theta$$

A Cartesian coordinate system is established with the disc’s center as the origin, the positive *x*-axis extending vertically upward, and the positive *y*-axis extending horizontally left. The time-varying coordinates of the center of mass are denoted as $\;\left(x_{c},y_{c}\right)$. Within the angular interval $\left[\theta_{1},\theta_{2}\right]$, the radius is modified to $r\left(\theta \right)=R-\Delta R\left(\theta \right)$, where $\Delta R\left(\theta \right)$ is a known function describing the radial shortening; outside this interval, the radius remains R. The lever arm length is consequently given by the absolute value of the *x*-coordinate, $|x_{c}|$. To obtain the driving torque $M_{a2}$, it is necessary to find the effective lever arm length during translational motion.

(10) $$M_{a2}=mg\cdot \left|x_{c}\right|$$

In the polar coordinate system, the area of the disc is given by $A=\frac{1}{2}\int_{0}^{2\pi }{\left[r\left(\theta \right)\right]}^{2}d\theta$. Partitioning the integral into the modified and unmodified angular intervals, then substituting and simplifying, yields the expression of *A*. Similarly, the moments $M_{x}$ and $M_{y}$ can be obtained. The coordinates of the center of mass are then given by $x_{c}=M_{y}/A$ and $y_{c}=M_{x}/A$. Substituting the expressions into the former and simplifying leads to the expression for $x_{c}$.

Inserting Equations (9) and (10) into (8) fields that

(11) $$M_{a}=\frac{mg}{4\pi }\int_{\pi /2}^{3\pi /2}\left[R-∆R\left(\theta \right)\right]sin\theta d\theta +mg\cdot \frac{1}{\pi }\int_{\pi /2}^{3\pi /2}∆R\left(\theta \right)\cos\theta d\theta$$

where m denotes the total mass of mass blocks, g represents the gravitational acceleration, $\Delta R\left(\theta \right)$ represents the projected displacement from each mass block to the edge of the disc, and $\left[R-\Delta R\left(\theta \right)\right]sin\theta d\theta$ corresponds to the effective lever arm of the gravitational force at angular position *θ*.

Equation (11) captures the underlying physical mechanism: photothermally induced bending of a bilayer displaces its associated mass block inward by $\Delta R\left(\theta \right)$,

(12) $$∆R\left(\theta \right)=\kappa \left(\theta \right)-\sqrt{\kappa^{2}\left(\theta \right)-L^{2}}$$

Here, $\kappa \left(\theta \right)$ denotes the photothermally decreasing radius of curvature of the LCE layer. Preceding analysis of the temperature field indicates that each LCE bilayer contracts to a different extent depending on its local temperature. For simplicity, a linear dependence is assumed to alter *R* and θ, expressed as

(13) $$\kappa \left(\theta \right)=AT\left(\theta \right)$$

Here, A represents the curvature coefficient. The LCE’s intrinsic anisotropy, which primarily controls its differential photothermal contraction, is effectively captured in this study through the introduced contraction coefficient. Combining Equations (11)–(13), the total driving torque responsible for both self-spinning and translation can be expressed as

(14) $$Ma=\frac{mg}{\pi }\int_{\pi /2}^{3\pi /2}\left[AT\left(\theta \right)-\sqrt{{\left[AT\left(\theta \right)\right]}^{2}-L^{2}}\right]\left(\cos\theta -\frac{1}{4}\sin\theta \right)d\theta$$

Assume that the characteristic heat exchange time is significantly longer than the nematic–isotropic phase transition period. We can simplify the study by adopting this assumption through the dimensionless parameter. Equation (16) becomes

(15) $$\bar{M_{a}}=\frac{\bar{g}}{\pi }\int_{\pi /2}^{3\pi /2}\left[\bar{A}\bar{T}\left(\theta \right)-\sqrt{{\left[\bar{A}\bar{T}\left(\theta \right)\right]}^{2}-{\bar{L}}^{2}}\right]\left(\cos\theta -\frac{1}{4}\sin\theta \right)d\theta$$

Once $\bar{T}\left(\theta \right)$ is derived from Equations (6) and (7), $\bar{M_{a}}$ can be calculated. Finally, an exploration is conducted into the effects of $\bar{I_{0}}$, $\bar{g}$, $\bar{A}$, $\bar{L}$, $\alpha =\left[\theta_{1},\theta_{2}\right]$ and k on $\bar{M_{a}}$.

## 3. LCE-Based Disc Exhibiting Steady Self-Spin and Translation

Upon reaching steady self-spinning and translation, the system experiences zero angular acceleration, and its shape does not vary with time. In this equilibrium condition, the LCE-based disc is exposed to two diametrically opposed torques, the photothermally generated driving torque Ma and the resistive damping torque $M_{d}$, which are presented in Figure 1c.

### 3.1. Mechanics Underlying Steady Self-Spinning and Translation

To research the self-spinning and translational motion under continuous illumination, appropriate dimensionless parameter values must be selected. Drawing on earlier experimental findings [77,78,79], the corresponding parameters and value can be seen in Table 1.

**Figure 3:**
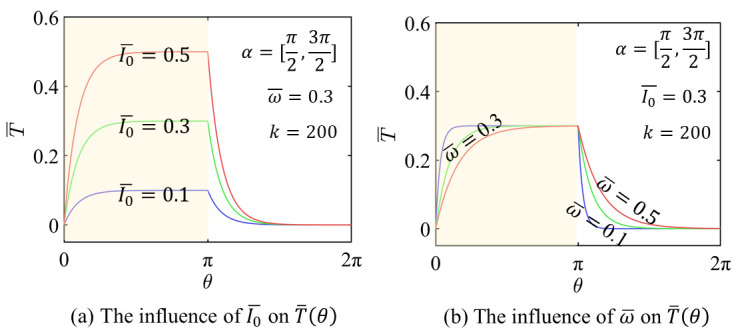

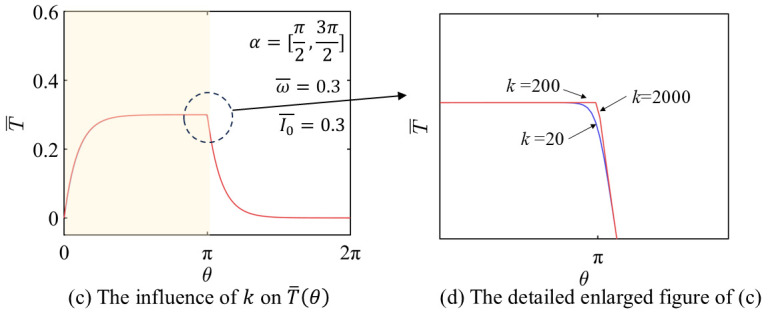
The temperature distribution $\bar{T}\left(\theta \right)$.

The illuminated portion of the disc is highlighted in yellow in each sub-figure, shown in Figure 3. In Figure 3a, $\bar{T}\left(\theta \right)$ exhibits a significant increase as $I_{0}$ keeps rising. In particular, higher values of $I_{0}$ cause LCE layers to absorb more heat, which clearly raises the $\bar{T}\left(\theta \right)$. Figure 3b shows that $\bar{T}\left(\theta \right)$ increases with decrease of $\bar{\omega }$, even though the peak temperature remains almost constant. A smaller $\bar{\omega }$ leads to a longer exposure duration for each bilayer, allowing more heat accumulation; beyond a certain point, however, the peak temperature saturates and does not rise further. In Figure 3c, the transition parameter *k* = 20, 200 and 2000 and is seen to have minimal impact on the temperature profile. As shown in Figure 3d, the detailed enlarged figure of (c), a slight effect is detected in the vicinity of the light–dark boundary.

**Figure 4:**
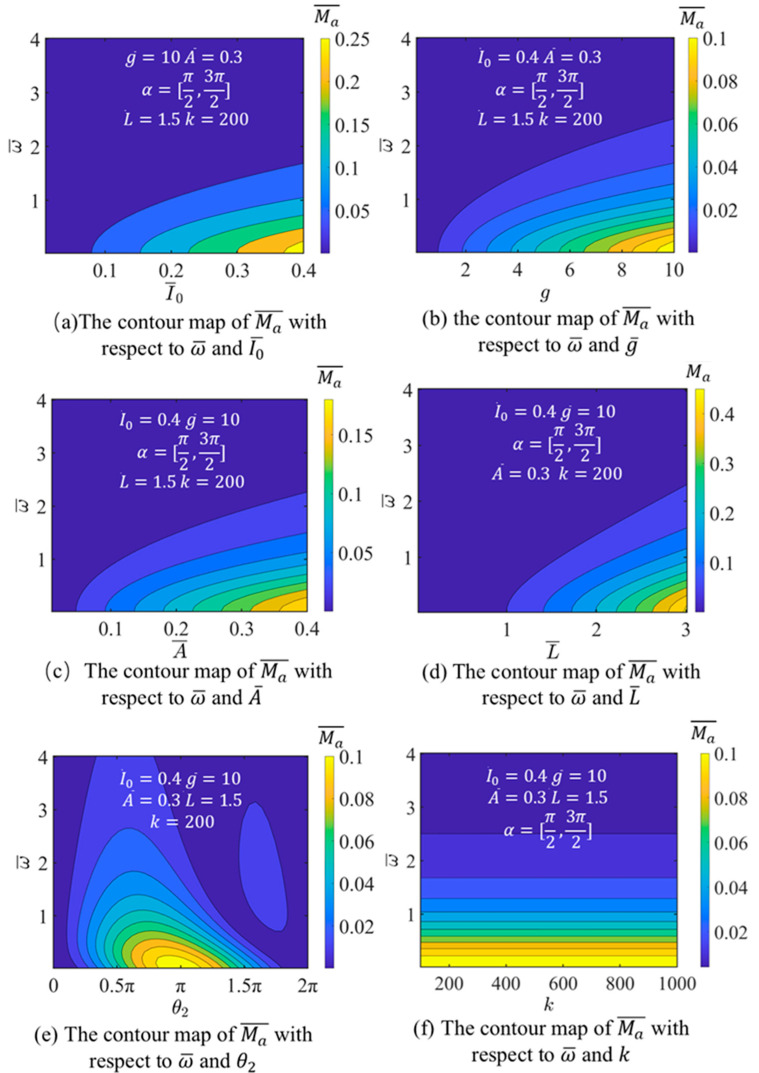
Influence of $\bar{I_{0}}$, $\bar{g}$, $\bar{A}$, $\bar{L}$, $\theta_{2}$ and k on driving self-spinning moment $\bar{M_{a}}$.

Figure 4 illustrates the variation in the dimensionless driving torque $\bar{M_{a}}$ with specific values noted in corresponding subfigure. From Figure 4a–d, we can know that the $\bar{I_{0}}$, $\bar{A}$, and $\bar{L}$ represent the disc’s photothermal energy uptake and its characteristics of structural response, while $\bar{g}$ accounts for the role of gravity in driving rotation. The consequences consistently show that $\bar{M_{a}}$ rises with increases in $\bar{I_{0}}$, $\bar{g}$, $\bar{A}$ and $\bar{L}$.

Figure 4e presents that the $\bar{M_{a}}$ increases at first and then decreases. When $\theta_{2}$ is small, $\bar{M_{a}}$ increases as a result of the contraction of specific LCE layers. As $\theta_{2}$ continues to increase, more LCE layers contract, further influencing $\bar{M_{a}}$. At this point, the self-spinning and translational torques generated by different LCE layers counteract each other, leading to a decrease in $\bar{M_{a}}$.

Figure 4f reveals that the switching coefficient k exerts negligible influence on $\bar{M_{a}}$; therefore, its effect is not considered further in the analysis. In all subsequent analyses, k is held fixed at 200. Moreover, Figure 4 further illustrates that $\bar{M_{a}}$ decreases as the dimensionless angular velocity $\bar{\omega }$ increases.

Under self-spinning and translation, the radiating spokes are subjected to $M_{a}$ and damping rotating moment $M_{d}$, as shown in Figure 1d. At the equilibrium condition $M_{a}=M_{d}$, a steady-state angular velocity solution for self-spinning emerges. If $M_{a}>M_{d}$, the strain in the LCE radiating spokes intensifies, thereby reducing the self-spinning angular velocity. In contrast, when $M_{a}<M_{d}$, the angular velocity ascends. Ultimately, the spoke’s angular velocity converges to a stabilized value. Furthermore, for LCE radiating spokes maintaining steady self-spinning, $M_{d}$ may be treated as invariant. The dimensionless rotational moment equilibrium is expressed as

(16) $$\bar{M_{a}}=\bar{M_{d}}$$

where $\bar{M_{d}}=\frac{M_{d}\tau^{2}}{mR^{2}}$.

The solution process of the above equation is as follows: substituting $\bar{I_{0}}$, ω and α into Equation (6) and $\bar{T}\left(0\right)=\bar{T}\left(2\pi \right)$ to calculate $T\left(\theta \right)$. Substituting the results into Equation (17), $M_{a}$ can be obtained. The LCE radiating spokes are regarded as being in a stable self-spinning state when $M_{a}$ meets Equation (18).

### 3.2. Limit Conditions of Self-Spinning and Translation

From the discussion above, we describe the procedure for evaluating the dimensionless driving torque’s critical limit, represented by ${\bar{M}}_{a}^{lim}$. It follows that ${\bar{M}}_{a}^{lim}$ can be determined as the system approaches the initiation of self-spinning and translation. The temperature distribution $\bar{T}\left(\theta \right)$ can be expressed in piecewise form as follows:

(17) $$\left\{\begin{array}{l} \bar{T}\left(\theta \right)=\bar{I_{0}},\theta_{1}\leq \theta \leq \theta_{2}\\ \bar{T}\left(\theta \right)=0\;,0<\theta <\theta_{1}\cup \theta_{2}<\theta <2\pi \end{array}\right.$$

Using Equations (14)–(17), we have

(18) $${\bar{M}}_{a}^{lim}=\frac{\bar{g}}{\pi }(\sqrt{{\left(\bar{A}\bar{I_{0}}\right)}^{2}-{\bar{L}}^{2}}+{\bar{L}}^{2}-\bar{A}\bar{I_{0}})(\sin\theta_{1}-\sin\theta_{2})\;+\frac{1}{4}\left(\cos\theta_{1}-\cos\theta_{2}\right)$$

**Figure 5:**
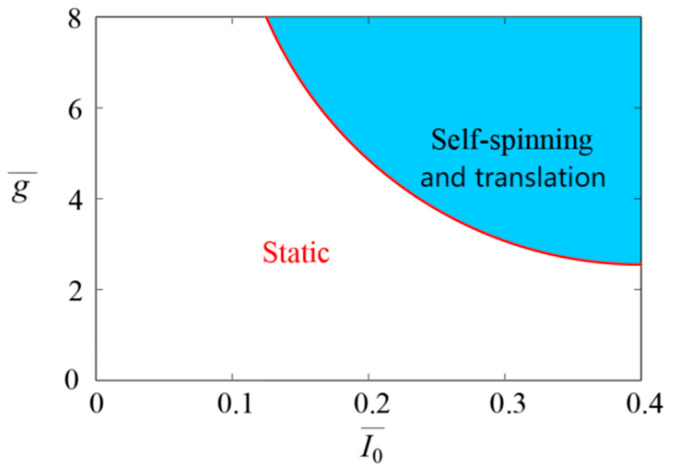
The relationship between $\bar{I_{0}}$ and $\bar{g}$.

The critical conditions for the steady self-spinning and translation can be determined using Equation (15). The critical condition is influenced by a number of critical parameters, such as $\bar{I_{0}}$, $\bar{g}$, $\bar{A}$, $\bar{L}$, $\theta_{1}$ and $\theta_{2}$. Figure 5 illustrates that the blue region corresponds to the parameter set where the LCE-based disc exhibits self-spinning and translation. All parameter combinations lying outside this region remain static with no sustained motion.

In practical applications, static friction generally exceeds dynamic friction, and the damping rotational torque $M_{d}$ arises from static friction. Increasing the damping coefficient will weaken self-oscillation amplitude. In order to improve the efficiency of light energy collection, it is recommended to reduce the damping coefficient in engineering practice.

## 4. Steady Translational and Self-Rotational Velocities

Building on a well-established theoretical framework, a study is conducted on how system parameters affect the steady angular velocity. Specifically, the steady-state angular velocity *ω* of the self-spinning and translation system are modulated by variations in critical parameters.

### 4.1. The Influence Originating from Heat Flux

**Figure 6:**
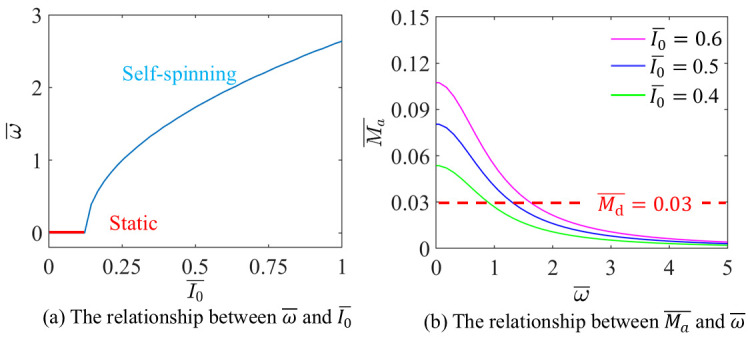
The influence of $\bar{I_{0}}$ on the self-spinning characteristics.

The influence of $\bar{I_{0}}$ on the $\bar{\omega }$ are discussed, with $\bar{g}=10$, $\bar{A}=0.3$, $\bar{L}=1.5$, $\alpha =\left[\frac{\pi }{2},\frac{3\pi }{2}\right]$ and $\bar{M_{d}}=0.03$, as shown in Figure 6.

Figure 6a illustrates that when $\bar{I_{0}}>0.1221$, with the increase of $\bar{I_{0}}$, $\bar{\omega }$ increases. The LCE-based disc remains static when $\bar{I_{0}}<0.1221$, which is in consistent with the critical value obtained by solving Equation (18).

As shown in Figure 6b, the $\bar{M_{a}}$ decreases with the increases of $\bar{\omega }$. The points where the dashed and solid lines intersect signify the initiation of steady self-spinning and translation. The data in Figure 6 illustrates fundamental trade-off between optical drive and damping resistance; while sufficient light input is essential to trigger and sustain motion, overly high damping can stifle self-spinning and translation. Consequently, increasing optical power or material absorption can effectively raise the operational speed, but concurrently, reducing the impact of damping contributions, including examples like friction-induced or viscous-loss-related contributions, is vital for achieving high efficiency and stable operation in devices actuated by light.

### 4.2. The Influence Originating from Gravitational Acceleration

**Figure 7:**
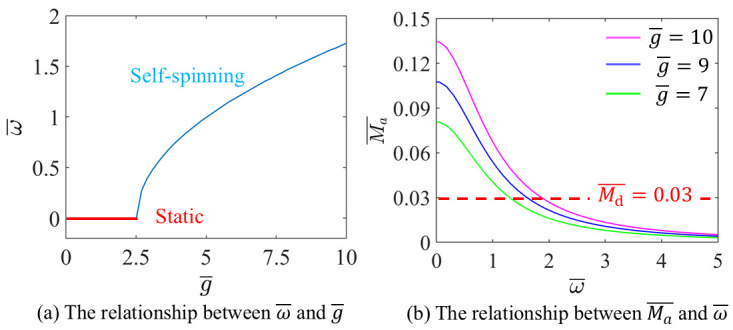
The influence of $\bar{g}$ on the self-spinning characteristics.

The influence of $\bar{g}$ on the $\bar{\omega }$ are discussed, with $\bar{I}=0.4$, $\bar{A}=0.3$, $\bar{L}=1.5$, $\alpha =\left[\frac{\pi }{2},\frac{3\pi }{2}\right]$ and $\bar{M_{d}}=0.03$, as shown in Figure 7.

Figure 7a illustrates that when $\bar{g}>2.5115$, with the increase of $\bar{g}$, $\bar{\omega }$ increases. The LCE-based disc remains static when $\bar{g}<2.5115$, which corresponds to the critical value derived from Equation (18).

Figure 7b shows that the $\bar{M_{a}}$ decreases with the increases of $\bar{\omega }$. The points where the dashed and solid lines intersect signify the initiation of steady self-spinning and translation. The data in Figure 7 illustrates that the driving torque is fundamentally generated by gravitational action on the asymmetrically displaced mass blocks: augmenting the block mass enhances torque output and promotes self-spinning and translation motion, whereas damping forces counteract this impetus, constraining the maximum attainable velocity. Consequently, system performance can be modulated through deliberate mass selection: although greater mass favors more stable and sustained self-spinning and translation, excessively heavy blocks may compromise structural integrity and accelerate material fatigue. Thus, achieving an optimal balance between inertial augmentation and damping mitigation is critical for developing high-efficiency, long-lasting light-actuated spinning systems.

### 4.3. The Influence Originating from Contraction Coefficient

**Figure 8:**
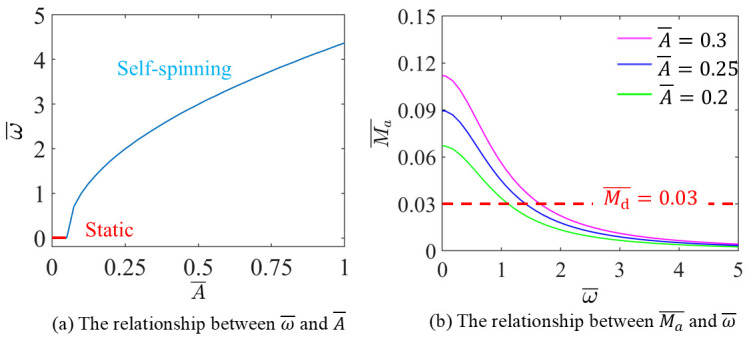
The influence of contraction coefficient $\bar{A}$ on the self-spinning characteristics.

The influence of heat flux $\bar{A}$ on the $\bar{\omega }$ are discussed, with $\bar{I}=0.4$, $\bar{g}=10$, $\bar{L}=1.5$, $\alpha =\left[\frac{\pi }{2},\frac{3\pi }{2}\right]$ and $\bar{M_{d}}=0.03$, as shown in Figure 8.

Figure 8a illustrates that when $\bar{A}>0.0505$, with the increase of $\bar{A}$, $\bar{\omega }$ increases. The LCE-based disc remains static when $\bar{I_{0}}<0.0505$, which corresponds to the critical value derived from Equation (18).

Figure 8b shows the variation of $\bar{M_{a}}$ with $\bar{\omega }$. With increases of $\bar{\omega }$, $\bar{M_{a}}$ decreases. The data in Figure 8 signifies the initiation of steady self-spinning. The findings establish contraction coefficient serves as a direct metric for the photothermal-to-mechanical energy conversion efficiency. A higher coefficient amplifies bilayer curvature, resulting in increased mass displacement and, consequently, a more substantial driving torque. From the viewpoint of practicality, focusing on LCE materials with outstanding contraction efficiency as a top concern is a straightforward strategy to boost self-spinning and translational performance. However, in practical implementations, excessive contraction can induce elevated internal stresses or hasten material degradation. Therefore, achieving an optimal trade-off between high contractility and long-term operational resilience is paramount for engineering stable, light-driven spinning systems.

### 4.4. The Influence Originating from Length of LCE

**Figure 9:**
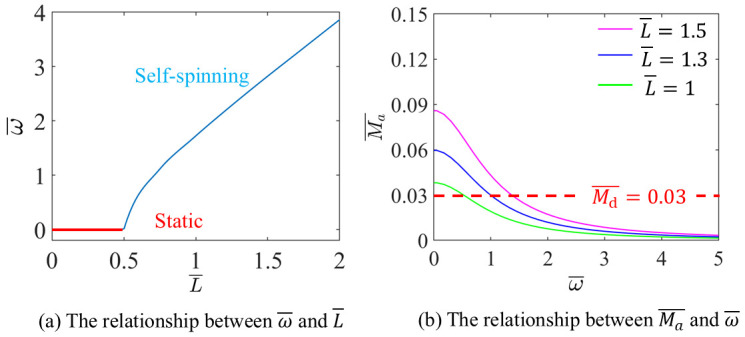
The influence of $\bar{L}$ on the self-spinning characteristics.

The influence of $\bar{L}$ on the $\bar{\omega }$ are discussed, with $\bar{I}=0.4$, $\bar{g}=10$, $\bar{A}=0.3$, $\alpha =\left[\frac{\pi }{2},\frac{3\pi }{2}\right]$ and $\bar{M_{d}}=0.03$, as shown in Figure 9.

Figure 9a illustrates that when $\bar{L}>0.0511$, with the increase of $\bar{L}$, $\bar{\omega }$ increases. The LCE-based disc remains static when $\bar{L}<0.0511$, which corresponds to the critical value derived from Equation (15).

Figure 9b shows $\bar{M_{a}}$ decreases with increases in $\bar{\omega }$. The points where the dashed and solid lines intersect signify the initiation of steady self-spinning and translation. The data in Figure 9 illustrates that while a greater LCE length amplifies the flexural amplitude during bending, it concurrently elevates the intralaminar internal stress and strain. To maintain the actuated curvature, a higher illumination input becomes necessary, thereby diminishing the net velocity under fixed input conditions. Practically, this underscores the critical need to optimize the LCE length: an insufficient length fails to produce adequate torque for motion initiation. However, an excessively long length increases the energy required for deformation and reduces system efficiency. Consequently, identifying a balanced LCE length is fundamental to realizing efficient and stable self-spinning in practical LCE-based devices.

## 5. Conclusions

As a theoretical framework describing an LCE-based disc system that can maintain steady spinning and translational motion, asymmetric illumination is proposed in this study. Unlike conventional self-oscillating systems that often suffer from vibrations, friction, and noise, the mechanism proposed in this study enables smooth and continuous motion by harnessing photothermal contraction in the upper hemisphere of the disc. The present model generates both a driving torque for self-spinning and a net propulsive force for translation, allowing the system to move steadily along a track without complex control or external power modulation.

By confining illumination to the upper hemisphere of the disc, the present study induces spatially non-uniform photothermal contraction in the LCE bilayers. This asymmetry shifts the system’s center of mass dynamically and generates not only a continuous driving torque for self-spinning but also a net propulsive force for translation under gravity, which represents a distinct departure from conventional oscillatory LCE systems. Theoretical analysis confirms that the steady self-spinning and translational velocities can be effectively regulated by key system parameters, including parameters like the heat flux (light intensity), contraction coefficient of the LCE, the bilayer length, and the angular span of the illumination zone. This tunability offers a practical pathway for controlling motion characteristics in application-specific scenarios.

The system’s ability to maintain stable hybrid motion under simple constant lighting offers a practical alternative to traditional oscillatory soft actuators. It minimizes unwanted dynamic fluctuations and reduces reliance on external controllers, making it suitable for applications where precision, quiet operation, and energy autonomy are critical—such as in soft robotics, micro-transporters, and on-surface sensors. By adjusting illumination or structural parameters, the self-spinning and translational speeds can be tuned, providing flexibility in real-world scenarios. The present study not only offers a new design strategy for creating efficient and quiet soft actuators but also opens a path toward wirelessly controlled, multi-functional soft machines capable of operating in noise- and vibration-sensitive environments.

## Figures and Tables

**Table 1:** Material properties and geometric parameters.

Parameter	Value	Unit
*A*	0~0.001	cm/K
*R*	5	cm
*L*	0.5~10	cm
$\theta_{1}$	0~2π	rad
$\theta_{2}$	0~2π	rad
$M_{d}$	0~0.001	N·m
$\tau_{0}$	0.001~0.1	s
$I_{0}$	0~2 × 10^3^	W
*K*	0~8	W/K
$\rho_{c}$	0.1	J/K
$T_{e}$	300	K
*g*	0~50	$\frac{m}{s^{2}}$
*m*	0~0.01	kg
*k*	10~1000	/
$\bar{I_{0}}$	0~0.8	/
$\bar{g}$	0~10	/
$\bar{A}$	0~1.5	/
$\bar{L}$	0.1~2	/
$\theta_{2}$	0~2π	/
$\bar{M_{d}}$	0~0.1	/

## Data Availability

The original contributions presented in this study are included in the article. Further inquiries can be directed to the corresponding author.
